# Risk Factors Associated With Positive Intraoperative Valve Tissue Cultures and the Subsequent Impact on Infective Endocarditis Management and Outcomes

**DOI:** 10.1093/ofid/ofag568

**Published:** 2026-09-19

**Authors:** Nirjan Bhattarai, Ryan W Stevens, Larry M Baddour, Daniel C DeSimone, Supavit Chesdachai, Joya-Rita Hindy, Ross A Dierkhising, Dan Ilges

**Affiliations:** Department of Pharmacy, Mayo Clinic, Rochester, Minnesota, USA; Department of Pharmacy, Mayo Clinic, Rochester, Minnesota, USA; Division of Public Health, Infectious Diseases and Occupational Medicine, Department of Medicine, Mayo Clinic, Rochester, Minnesota, USA; Department of Cardiovascular Medicine, Mayo Clinic, Rochester, Minnesota, USA; Division of Public Health, Infectious Diseases and Occupational Medicine, Department of Medicine, Mayo Clinic, Rochester, Minnesota, USA; Department of Cardiovascular Medicine, Mayo Clinic, Rochester, Minnesota, USA; Division of Public Health, Infectious Diseases and Occupational Medicine, Department of Medicine, Mayo Clinic, Rochester, Minnesota, USA; Department of Cardiovascular Medicine, Mayo Clinic, Rochester, Minnesota, USA; Division of Public Health, Infectious Diseases and Occupational Medicine, Department of Medicine, Mayo Clinic, Rochester, Minnesota, USA; Division of Clinical Trials and Biostatistics, Mayo Clinic, Rochester, Minnesota, USA; Department of Pharmacy, Mayo Clinic Arizona, Phoenix, Arizona, USA

**Keywords:** antibiotic stewardship, infective endocarditis, postoperative antibiotic duration, preoperative antibiotic duration, surgical valve replacement

## Abstract

**Background:**

Cardiac valve tissue obtained during the surgical management of infective endocarditis (IE) and sent for culture may influence postoperative antibiotic therapy. We sought to identify clinical and microbiologic risk factors associated with positive valve cultures (PVCs) and IE relapses in a cohort of patients undergoing surgical management.

**Methods:**

This is a retrospective, multicenter study of patients with surgical management during the acute course (<42 days) of IE-directed antibiotic therapy. The rate of PVC, IE relapse, and mortality were collected. Univariate and multivariable logistic regressions were performed to identify risk factors associated with PVC.

**Results:**

Of 113 patients included, 36 (31.9%) had PVCs. Univariate and multivariable analyses showed that preoperative antibiotic duration and pathogens were associated with PVC. The median number of days of preoperative antibiotics was 6.5 (interquartile range [IQR], 4.0–11.0) for patients with PVCs compared to 13.0 (IQR, 8.0–21.0) for those with negative valve cultures (NVCs). In a multivariable model, preoperative antibiotic administration decreased the risk of PVC (odds ratio [OR], 0.85 per day [95% CI, .77–.91]). Enterococcal IE increased the risk of PVC compared to streptococcal IE (OR, 4.16 [95% CI, 1.07–17.48]). Of note, 33 (63.2%) patients with NVCs received a median of 8 (IQR, 5–18.5) days of avoidable intravenous antibiotics per patient. Only 1 IE relapse was identified and occurred in a patient with NVCs.

**Conclusions:**

Both preoperative antibiotic duration and causative pathogen were associated with PVC. IE relapses were rare and unrelated to PVC. There may be an opportunity to shorten postoperative antibiotic durations following surgical management in patients with IE.

Infective endocarditis (IE) is a life-threatening disease with high morbidity and mortality, often requiring surgical intervention [1, 2]. Surgical management with debridement and valve repair or replacement is indicated in up to 50% of cases, most often secondary to valvular dysfunction or uncontrolled infection, or for prevention of septic emboli [3].

Historically, surgical valve replacement or repair for IE was delayed, when possible, to facilitate a period of preoperative antibiotic administration prior to surgical intervention to “better sterilize” infected tissue. More recently, this approach has shifted, favoring earlier interventions to reduce embolic events and mortality [4, 5].

The incidence of valve tissue culture positivity is approximately 25% [6–8]. Early surgical intervention results in a shorter duration of preoperative antibiotics, which may increase the likelihood of positive valve cultures (PVCs) [9]. This is accompanied by a conceptual concern that implantation of prosthetic material in an infected surgical field increases the infection risk of the newly placed prosthetic valve leading to IE relapse, particularly with biofilm-forming organisms. Thus, determining the optimal timing of surgery is complex, requiring integration of multiple clinical factors and coordinated decision making by a multidisciplinary team including cardiology, radiology, cardiovascular surgery, and infectious diseases specialists [10]. Recurrence of IE following surgical valve repair or replacement is estimated to occur in 2%–10% of the cases [11–15]. Recurrence refers to both reinfection and relapse. Reinfection is characterized by isolation of a different microorganism, whereas relapse refers to a repeat episode caused by the same microorganism [16]. Reinfection accounts for most recurrences, with relapse detected in approximately 20% of recurrent cases per the European Infective Endocarditis (EURO-ENDO) registry [17].

In response to the risk of IE relapse, some have posited that the results of valve cultures should be used to determine postoperative antibiotic duration. There is a paucity of data assessing whether extending antibiotic duration postoperatively in patients with PVCs improves outcomes, as existing studies either exclude these patients or include limited numbers [18–21]. In the absence of prospective clinical trial data, the 2015 American Heart Association (AHA) Scientific Statement recommended restarting the antibiotic course after valve surgery if operative tissue cultures are positive [22]. Similarly, the 2023 European Society of Cardiology (ESC) IE guidelines recommend, based on expert opinion, 6 weeks of antibiotic therapy following valve surgery with PVC [3]. A 2020 survey of infectious disease physicians reported that 86% of respondents prescribed 6 weeks of antibiotics postsurgical valve replacement or repair in setting of PVC with methicillin-resistant *Staphylococcus aureus* native valve endocarditis. However, for patients with negative valve cultures (NVCs), the responses were varied, with 20% still opting to “restart the clock” and treating for 6 weeks postoperatively [23]. Extending antibiotic duration following source control in this setting is contrary to conventional wisdom in infectious diseases, where source control typically facilitates a shorter treatment duration. Thus, a better understanding of the relationship between operative valve cultures, antibiotic treatment duration, and relapse risk is warranted.

The current investigation aims to characterize the incidence of PVC in patients undergoing surgical valve replacement or repair for IE during the acute antibiotic treatment course. Subsequently, we aim to identify clinical and microbiologic risk factors associated with PVC, describe postoperative antibiotic durations in those with both PVC and NVC, and report characteristics associated with IE relapse and all-cause mortality.

## METHODS

### Study Design

This is a retrospective, noninterventional, multicenter study of patients who underwent surgical valve replacement or repair while on active treatment for IE within the Mayo Clinic Enterprise. Adult patients (≥18 years) with active prosthetic or native valve IE using modified Duke criteria who underwent surgical valve replacement/repair prior to completion of the acute antibiotic course between 1 January 2018 and 31 December 2025 were eligible for inclusion. Included patients were required to have valve tissue cultures and demonstrate echocardiographic and/or radiographic (positron emission tomography–computed tomography) evidence of IE with blood cultures positive for *Staphylococcus*, *Streptococcus*, *Enterococcus*, *Abiotrophia*, *Gemella*, or *Granulicatella* species during the same hospital admission [24]. Patients were excluded if they declined the Minnesota Research Authorization (as per Minnesota Statue 144.295), completed their standard course of antibiotics prior to the date of surgery (≥42 days), or were pregnant or incarcerated [25]. Valve tissue cultures were collected in the operating room and processed as per local microbiology laboratory standard operating procedures. The study was approved by the Mayo Clinic Institutional Review Board with a waiver of informed consent (protocol no. 25-009601).

### Data Collection

Patient characteristics, valve tissue Gram stain, cultures, histopathologic results, postoperative antibiotic duration, and follow-up echocardiography were collected from electronic medical records. Variables including preoperative antibiotic duration, presence of perioperative fever, date of blood culture clearance, surgery duration, intensive care unit (ICU) admission, vegetation size, perivalvular abscess, risk factors associated with IE, number and location of septic emboli, and pathogen were collected.

Risk factors associated with IE included previously placed mechanical or bioprosthetic cardiac valve, previous episode of IE, valvular heart disease, or presence of a cardiac implantable electronic device (CIED). Alanine aminotransferase (ALT), aspartate aminotransferase (AST), alkaline phosphatase (ALP), *Clostridioides difficile* infection during treatment, complications secondary to peripherally inserted central lines (eg, bloodstream infection, pain/discomfort around the line insertion site), and other reported adverse drug events were also collected. Factors considered potentially influenced by valve tissue culture positivity, including relapse, mortality (1 year post–valve replacement or repair), postoperative antibiotic duration, and the decision to start suppressive antibiotic therapy after the acute treatment course was completed, were collected. The severity-weighted Charlson Comorbidity Index (CCI) was used to approximate comorbidity burden for individual patients [26]. Day 1 of therapy was defined as the first day of antibiotic therapy with in vitro activity against the isolated pathogen. Perioperative fever was defined as body temperatures ≥38.0°C 48 hours before and after the date of surgery. Hepatic impairment was defined as ALT ≥3×, AST ≥3×, or ALP ≥5× the upper limit of normal while on active antibiotic therapy for IE [27]. Data were extracted from the medical record using a structured data collection tool within REDCap (Research Electronic Data Capture, Vanderbilt University) hosted at Mayo Clinic [28].

### Outcomes

The primary outcomes included both rate and factors associated with PVC. Gram stain and/or polymerase chain reaction (PCR) positivity of operative valve specimens were evaluated separately and not considered surrogates for valve tissue culture positivity. Secondary outcomes included incidence of IE relapse and all-cause mortality occurring within 1 year after the date of surgery. Relapse was defined as IE caused by the same organism within 1 year after surgical valve replacement or repair and following successful completion of postoperative antibiotic treatment. Given the aims of the study, reinfection (ie, repeated infection with a different microorganism) was not collected. All patients were included in the primary outcome. However, people who inject drugs (PWID), those without 1-year follow-up echocardiography, those who had antibiotics extended for a non-IE indication, and those who did not survive the acute antibiotic course were excluded from the secondary outcome of relapse. Follow-up time for mortality was based on the most recent contact date for each patient.

### Statistical Analysis

Baseline characteristics were summarized as median (interquartile range [IQR]) for numeric variables and frequency (%) for categorical variables. Baseline group comparisons were performed using Wilcoxon rank-sum tests for numeric variables and Pearson χ^2^ test for categorical variables. Logistic regression models were fit for the outcome of valve tissue culture positivity, and a multivariable model was created using a priori selected predictors. Kaplan–Meier methodology was used to compare groups for 1-year, postoperative, all-cause mortality. *P* values <.05 were considered statistically significant. R statistical software version 4.5.1 (R Core Team [2025], R Foundation for Statistical Computing, Vienna, Austria, https://www.R-project.org) was used for the analysis, including the tidyverse, summarytools, arsenal, broom, survival, and ggsurvfit R packages.

## RESULTS

### Baseline Characteristics

Overall, 158 patients were screened, and 113 met inclusion criteria for the primary outcome. The sole reason for exclusion was the completion of antibiotic therapy prior to surgical valve replacement/repair (n = 45) (Figure 1). The median age was 60 (IQR, 48–70) years, and the majority of patients were male (73.5%). From the total cohort, 35 (31.3%) were Gram stain positive. Valve cultures were positive in 36 (31.9%) patients. A comparison of patient, infection, and surgical factors between those with PVCs and NVCs is listed in Table 1. All patients had an infectious diseases consultation during their index hospitalization. The 2 groups were statistically similar with respect to weight, CCI score, PWID status, ICU admission within 24 hours of hospital admission, valve(s) involved, vegetation size, presence of perivalvular abscess, risk factors for IE, surgery type, indication for surgery, CIED status, and embolic events. The most common pathogens were streptococci (n = 47), staphylococci (n = 46), and enterococci (n = 15). The remaining cases were due to *Abiotrophia* (n = 2), *Gemella* (n = 2), and *Granulicatella* species (n = 1) (Table 1). Broad-range PCR screening was done from valve tissue of 24 (20.3%) patients, of which 20 (83.3%) were positive.

**Figure 1.**
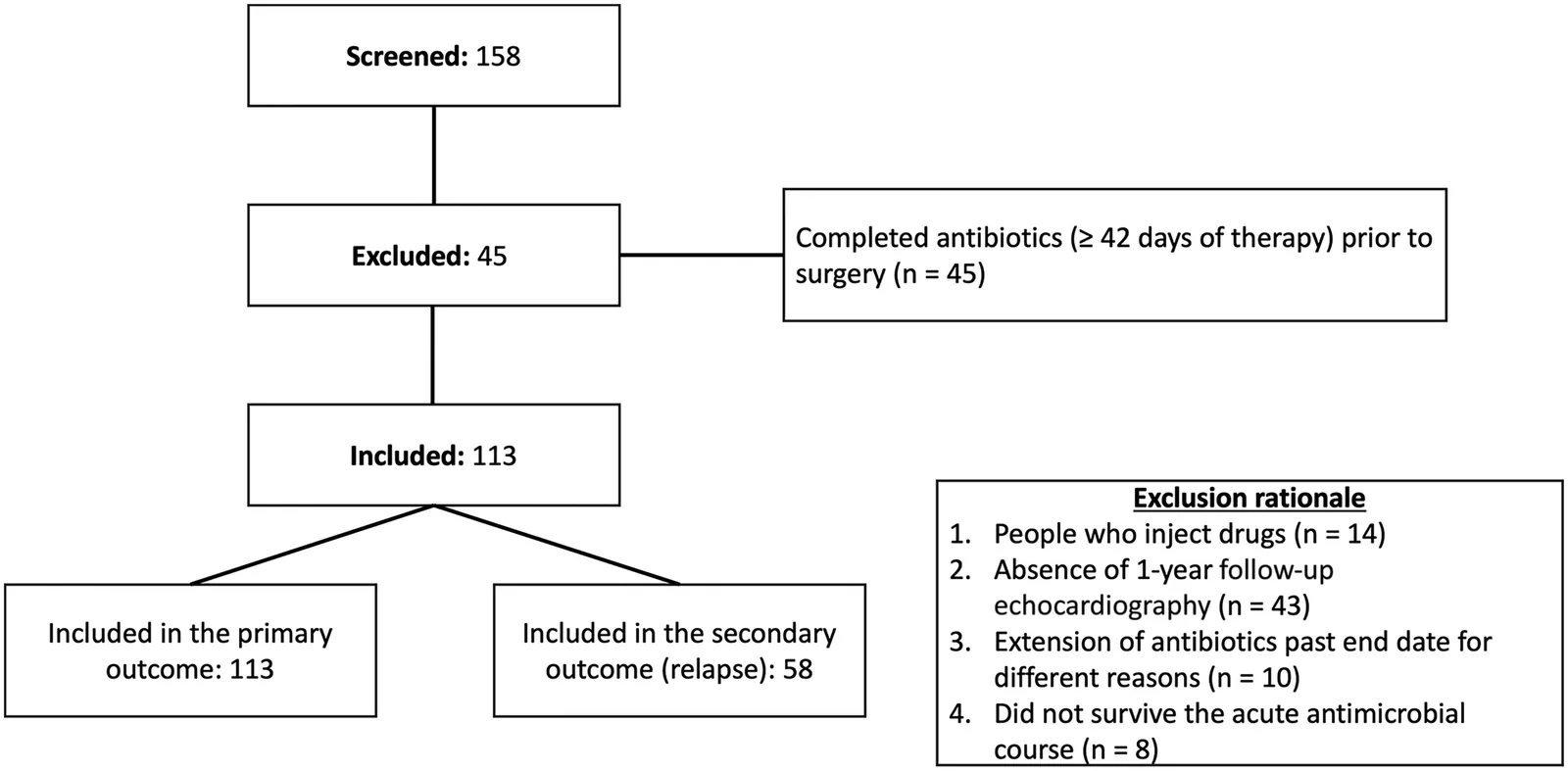
Inclusion/exclusion flowchart.

**Table 1.** Baseline Characteristics

Variable	NVC (n = 77)	PVC (n = 36)	Full Cohort (N = 113)	*P* Value
Female sex	22 (28.6%)	8 (22.2%)	30 (26.5%)	.476
Age, y	58.0 (48.0–69.0)	61.5 (49.2–71.2)	60.0 (48.0–70.0)	.299
Body weight, kg	87.3 (75.2–99.9)	88.7 (72.1–106.5)	87.3 (74.7–101.0)	.772
Body mass index, kg/m^2^	27.8 (24.7–32.5)	28.4 (24.8–34.8)	27.8 (24.7–33.3)	.844
Charlson Comorbidity Index score	5.0 (3.0–8.0)	4.0 (2.8–6.0)	5.0 (3.0–7.0)	.249
People who inject drugs	9 (11.7%)	3 (8.3%)	12 (10.6%)	.590
ICU admission within 24 h	24 (31.2%)	13 (36.1%)	37 (32.7%)	.750
Perioperative fever	21 (27.3%)	17 (47.2%)	38 (33.6%)	.036
Infected valve(s)				
Aortic valve	53 (68.8%)	24 (66.7%)	77 (68.1%)	.818
Mitral valve	29 (37.7%)	13 (36.1%)	42 (37.2%)	.874
Pulmonary valve	5 (6.5%)	2 (5.6%)	7 (6.2%)	.847
Tricuspid valve	8 (10.4%)	5 (13.9%)	13 (11.5%)	.587
Multiple valves (≥2)	17 (22.1%)	6 (16.7%)	23 (20.4%)	.506
Vegetation size, cm	1.4 (1.0–2.1)	1.5 (1.0–2.3)	1.5 (1.0–2.2)	.944
Presence of perivalvular abscess	24 (31.2%)	16 (44.4%)	40 (35.4%)	.169
PET-CT completed prior to surgery date	n = 18	n = 6	n = 24	
Evidence of intracardiac infection on preoperative PET-CT	17/18 (94.4%)	6/6 (100.0%)	23/24 (95.8%)	.555
Risk factors associated with IE				
Previous mechanical or bioprosthetic valve	28 (36.4%)	17 (47.2%)	45 (39.8%)	.272
Previous IE episode	9 (11.7%)	2 (5.6%)	11 (9.7%)	.306
Valvular heart disease	31 (40.3%)	10 (27.8%)	41 (36.3%)	.199
CIED (excluding leadless)	8 (10.4%)	6 (16.7%)	14 (12.4%)	.345
None of the above^a^	29 (37.7%)	15 (41.7%)	44 (38.9%)	.684
Surgery type				.190
Valve repair	1 (1.3%)	2 (5.6%)	3 (2.7%)	
Valve replacement	76 (98.7%)	34 (94.4%)	110 (97.3%)	
Valve type	n = 76	n = 34	n = 110	.876
Bioprosthetic	48 (63.2%)	22 (64.7%)	70 (63.6%)	
Mechanical	28 (36.8%)	12 (35.3%)	40 (36.4%)	
Indication for surgery^b^				
Heart failure	8 (11.1%)	3 (9.1%)	11 (10.5%)	.754
Recurrent emboli prevention	10 (13.9%)	4 (12.1%)	14 (13.3%)	.805
Uncontrolled infection	6 (8.3%)	2 (6.1%)	8 (7.6%)	.684
Vegetation size ≥1 cm	33 (45.8%)	20 (60.6%)	53 (50.5%)	.160
Perivalvular abscess	24 (33.3%)	12 (36.4%)	36 (34.3%)	.761
CIED status	n = 8	n = 6	n = 14	.417
Generator and leads retained	1 (12.5%)	1 (16.7%)	2 (14.3%)	
Generator removed, leads retained	0	0	0	
Both removed and replaced	5 (62.5%)	5 (83.3%)	10 (71.4%)	
Both removed, not replaced	2 (25.0%)	0 (0.0%)	2 (14.3%)	
Embolic events				
Central nervous system	40 (51.9%)	14 (38.9%)	54 (47.8%)	.195
Spleen	7 (9.1%)	9 (25.0%)	16 (14.2%)	.024
Renal	4 (5.2%)	2 (5.6%)	6 (5.3%)	.936
Pulmonary	17 (22.1%)	4 (11.1%)	21 (18.6%)	.163
Other	11 (14.3%)	5 (13.9%)	16 (14.2%)	.955
Duration of preoperative antibiotics, days	13.0 (8.0–21.0)	6.5 (4.0–11.0)	11.0 (6.0–18.0)	<.001
Bacteremia at time of surgery	3 (5.0%)	9 (29.0%)	12 (13.2%)	.001
If clearance prior to surgery and date is known, time from clearance to OR date, days^c^	11.0 (6.0–18.0)	3.0 (2.2–8.0)	9.0 (4.0–15.5)	<.001
Pathogens				
Staphylococci	29	17	46 (40.7%)	
*Staphylococcus aureus*	19/29 (65.5%)	10/17 (58.8%)	29/46 (63%)	
Non-*aureus* staphylococci^d^	10/29 (34.5%)	7/17 (41.2%)	17/46 (37%)	
Streptococci	37	10	47 (41.6%)	
Viridians group streptococci	24/37 (64.9%)	10/10 (100%)	34/47 (72.3%)	
Beta-hemolytic streptococci	11/37 (29.7%)	0/10 (0%)	11/47 (23.4%)	
Alpha-hemolytic streptococci	2/37 (5.4%)	0/10 (0%)	2/47 (4.3%)	
Enterococci	7	8	15 (13.3%)	
*Enterococcus faecalis*	6/7 (85.7%)	8/8 (100%)	14/15 (93.3%)	
*Enterococcus faecium*	1/7 (14.3%)	0/8 (0%)	1/15 (6.7%)	
*Abiotrophia* species	1	1	2/113 (1.8%)	
*Gemella* species	2	0	2/113 (1.8%)	
*Granulicatella* species	1	0	1/113 (0.9%)	
Intraoperative microbiology				
Gram stain, positive	14/76 (18.4%)	21/36 (58.3%)	35/112 (31.3%)	
PCR test done	20/77	4/36	24/113	
Positive broad-range PCR	17/20 (85.0%)	3/4 (75.0%)	20/24 (83.3%)	
Positive valve pathology	52/66 (78.8%)	32/35 (91.4%)	84/101 (83.2%)	

Data are reported as No. (%) or median (interquartile range).

Abbreviations: CIED, cardiac implantable electronic device; ICU, intensive care unit; IE, infective endocarditis; NVC, negative valve culture; OR, operating room; PCR, polymerase chain reaction; PET-CT, positron emission tomography–computed tomography; PVC, positive valve culture.

^a^Eight of the patients who had no cardiovascular risk factors were people who inject drugs.

^b^Eight patients in the total cohort had unknown indications for surgery per chart review.

^c^Number of patients where bacteremia clearance date prior to surgery was unknown: negative cultures (n = 20), positive cultures (n = 14), and full cohort (n = 34).

^d^There were 5 cases of *Staphylococcus lugdunensis* infection, of which 3 had PVCs.

### Predictors of Valve Tissue Culture Positivity

Univariate and multivariable analyses are shown in Table 2. The univariate analysis showed that fewer preoperative days of antibiotics and enterococcal infection (relative to streptococcal/other organisms) were associated with PVC. Other factors such as perivalvular abscess, prosthetic valve, and staphylococcal infection were not associated with PVC. In multivariable analysis, both preoperative days of antibiotics (odds ratio [OR], 0.85 per day [95% confidence interval {CI}, .77–.91]) and enterococcal infection (OR, 4.16 [95% CI, 1.07–17.48]) retained statistical significance. All other factors were not statistically significant.

**Table 2.** Logistic Regression Models for Culture Positivity

Variable	Univariate Analysis		Multivariable Analysis	
	OR (95% CI)	*P* Value	OR (95% CI)	*P* Value
Preoperative antibiotics, per day	0.85 (.78–.92)	<.001	0.85 (.77–.91)	<.001
Perivalvular abscess	1.77 (.78–4.01)	.171	1.51 (.55–4.15)	.423
Previous prosthetic valve	1.57 (.70–3.51)	.273	1.23 (.44–3.40)	.690
Organism (reference = streptococci/*Abiotrophia*/*Gemella*/*Granulicatella*)				
Staphylococci	2.18 (.90–5.47)	.087	2.79 (1.00–8.16)	.053
Enterococci	4.26 (1.27–14.84)	.019	4.16 (1.07–17.48)	.043

Abbreviations: CI, confidence interval; OR, odds ratio.

The median (IQR) preoperative duration of antibiotic treatment was 6.5 (4–11) and 13 (8–21) days for PVC and NVC, respectively (*P* < .001). Twelve patients were bacteremic at the time of surgery, with 9 (75%) of them having PVCs. Of those who cleared blood cultures prior to surgery, they were cleared for a median (IQR) of 6.5 (4.0–11.0) and 11 (6–21) days prior to surgery for PVC and NVC, respectively (*P* < .001) (Table 1, Figure 2).

**Figure 2.**
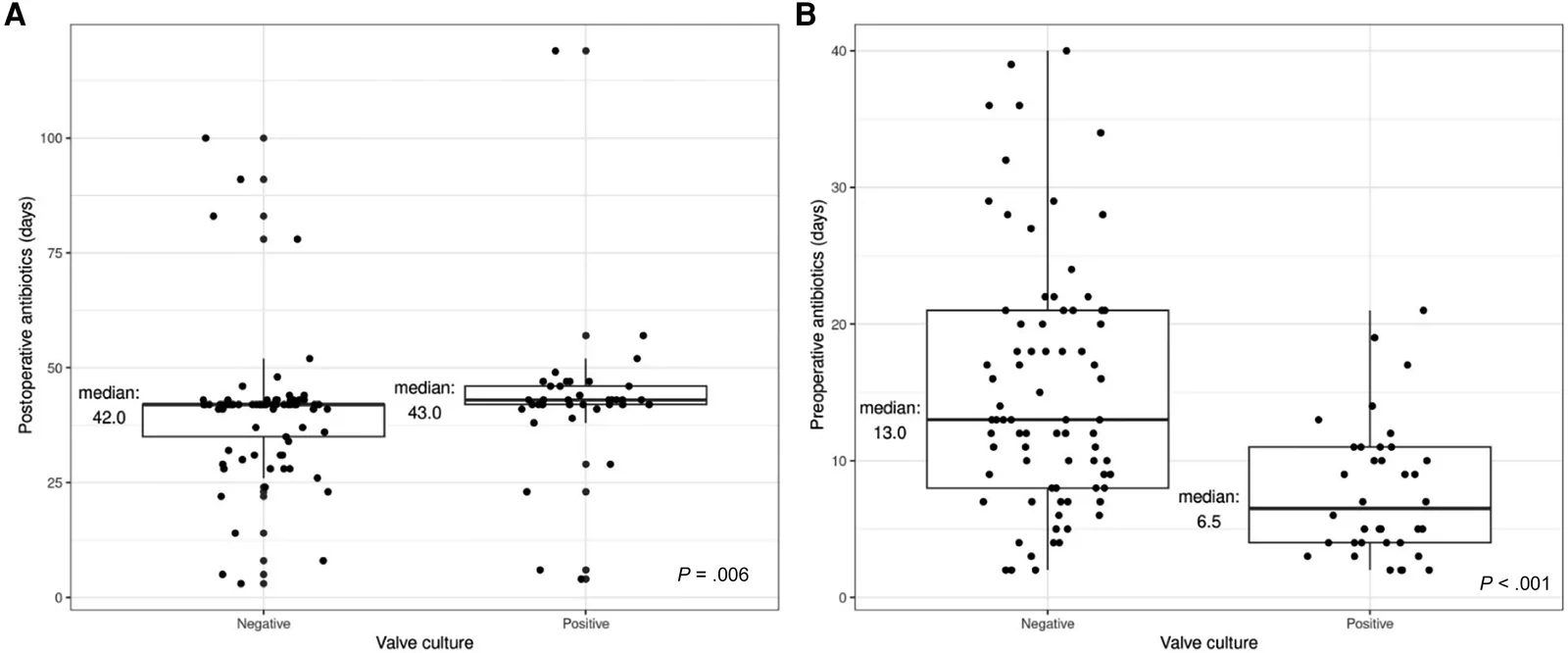
Boxplots of postoperative (A) and preoperative (B) days of antibiotics by valve culture positivity.

### IE Relapse and All-Cause Mortality

Of the 58 patients included in the secondary analysis of 1-year relapse, only 1 patient (1.7%) experienced relapse. All 113 patients were included in the analysis of postoperative all-cause 1-year mortality. A total of 19 (27.3%) patients died within 1 year, with no difference with respect to PVC (n = 3 [10.8%]) and NVC (n = 16 [33.5%]) (log-rank *P* = .20; Figure 3).

**Figure 3.**
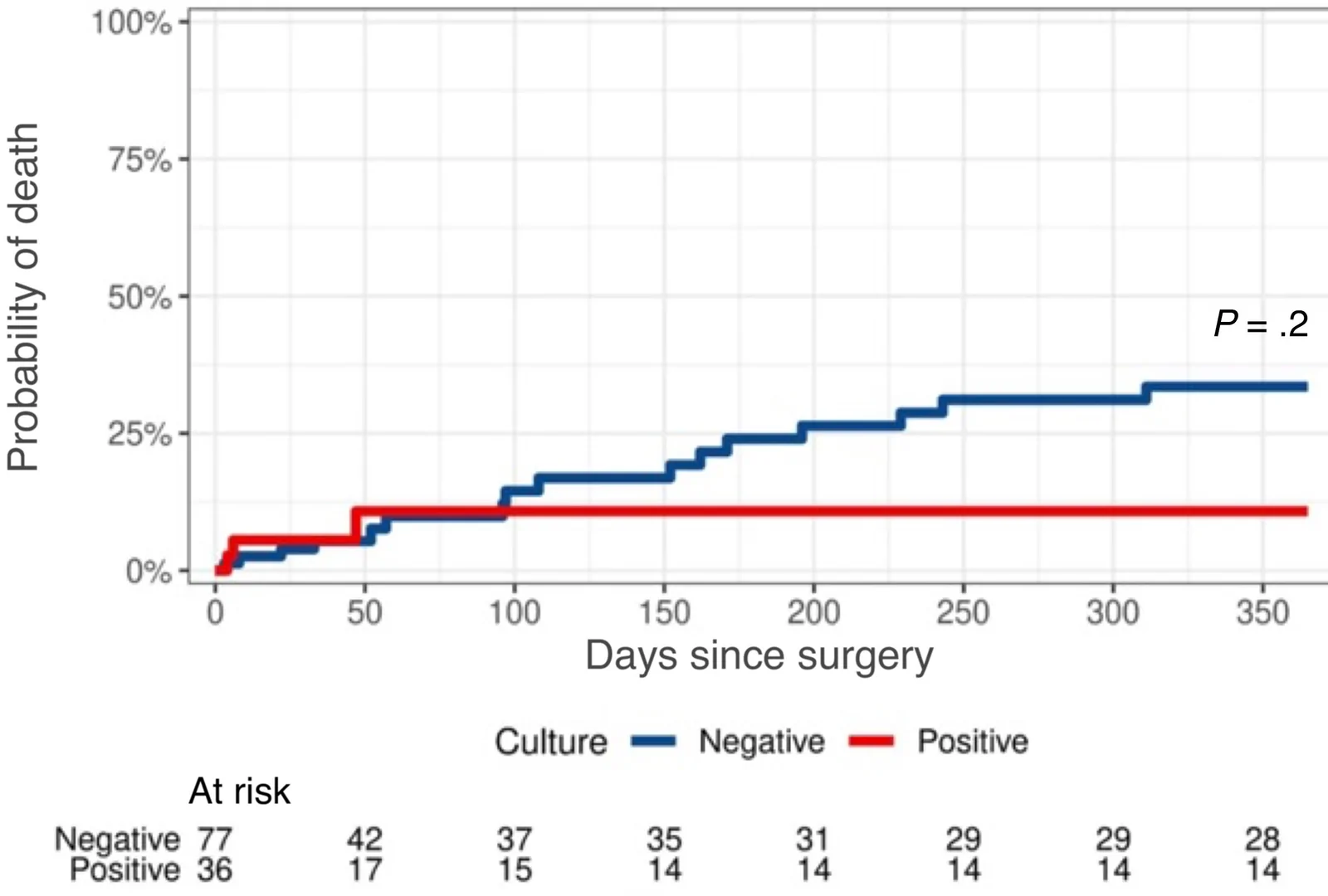
Kaplan–Meier curve for 1-year all-cause mortality by valve culture positivity.

### Postsurgical Antibiotic Therapy

The median (IQR) duration of postoperative antibiotics was 42 (35–42) and 43 (42–46) days for NVCs and PVCs, respectively (*P* = .006; Figure 2). Overall, 37 (32.7%) patients were placed on suppressive therapy. Of those who had PVCs, 13 (36.1%) were placed on suppressive therapy (Table 3). With regard to antibiotic adverse effects, hepatic impairment occurred in 28.3% of patients during active therapy, and there were 4 (3.5%) cases of *C difficile* infection and 5 (4.4%) line-related complications (Table 3).

**Table 3.** Antimicrobial Characteristics

Variable	NVC (n = 77)	PVC (n = 36)	Full Cohort (N = 113)	*P* Value
Antibiotic-related complications				
Hepatoxicity^a^	22 (28.6%)	10 (27.8%)	32 (28.3%)	.930
*Clostridioides difficile* infection	2 (2.6%)	2 (5.6%)	4 (3.5%)	.428
Catheter-related complications^b^	4 (5.2%)	1 (2.8%)	5 (4.4%)	.560
Other	10 (13.0%)	4 (11.1%)	14 (12.4%)	.778
None	44 (57.1%)	20 (55.6%)	64 (56.6%)	.874
Duration of postoperative antibiotics, days	42.0 (35.0–42.0)	43.0 (42.0–46.0)	42.0 (39.0–43.0)	.006
Placed on suppressive therapy (yes)	24 (31.2%)	13 (36.1%)	37 (32.7%)	.602

Data are reported as No. (%) or median (interquartile range).

Abbreviations: NVC, negative valve culture; PVC, positive valve culture.

^a^Defined as alanine aminotransferase ≥3×, aspartate aminotransferase ≥3×, or alkaline phosphatase ≥5× the upper limit of normal.

^b^Pain/discomfort (n = 3), bacteremia (n = 1), and superficial clot (n = 1).

Of the 57 patients who had negative blood cultures prior to surgery and had NVCs, 36 (63.2%) received >42 days of intravenous (IV) antibiotics from first negative blood cultures. Of those 36, 3 had antibiotics extended for reasons other than IE. Excluding those 3, if the date of blood culture clearance had been used as the first day of therapy, as per the 2015 AHA/Infectious Diseases Society of America (IDSA) recommendation, instead of “restart-the-clock” after surgery, a total of 437 antibiotic days would have been avoided, amounting to a median of 8 (IQR, 5–18.5) days of avoidable IV antibiotics per patient.

## DISCUSSION

In this multicenter, retrospective cohort of patients with surgical valve replacement or repair for management of IE, 31.9% had PVCs. Multivariable analysis revealed that each additional day of preoperative antibiotics may lower the odds of PVC by 15%. In addition, enterococcal IE had >4-fold higher odds of PVC as compared to that of streptococcal IE. The presence of perivalvular abscess or previous prosthetic valve was not significantly linked to a higher risk of valve tissue culture positivity. In secondary analyses, 1-year all-cause mortality occurred in 10.3% and 33.5% patients within the PVC and NVC cohorts, respectively. Having a PVC at the time of surgical repair or replacement was not associated with an increased risk of 1-year all-cause mortality or relapse as compared to NVC. However, only 1 IE relapse occurred (patient with NVC), and patients in the NVC cohort often received long postoperative durations that did not align with current AHA/IDSA recommendations. Thus, our mortality and relapse findings should be interpreted with caution and within the context of similar postoperative management strategies.

Few studies have examined patient-specific risk factors associated with PVC or relapse rates following surgical valve replacement or repair. Previously identified risk factors associated with PVC include shorter durations of preoperative antibiotic therapy (≤7 days), staphylococcal or enterococcal infection, large (>1 cm) vegetation size, and the presence of a preoperative fever. Additionally, although limited by small sample sizes and the ability to adjust for multiple confounders, studies have not linked PVC with higher rates of 1-year mortality or relapse [6–9]. Notably, a recent study reported an association between PVC and in-hospital mortality; however, these findings likely reflect a selection bias, as surgery may have been performed earlier for patients with more severe disease [29].

Our study is consistent with prior studies finding that longer preoperative antibiotic duration decreases the risk of PVC [6, 7, 30]. The PVC and relapse rates reported in this study are similar to those previously published, strengthening the evidence that the risk of relapse at 1 year seems to be low and unrelated to PVC [6, 18, 31]. Like prior studies, enterococcal and staphylococcal species tend to increase the risk of PVC compared to streptococcal species [7].

Both the 2015 AHA and 2023 ESC IE documents recommended counting “day 1” of therapy as the date of blood culture clearance in cases of NVC. Counting the surgery date as “day 1” of therapy is only recommended in PVC [3, 22]. However, in practice, the duration of therapy is often restarted from the day of surgery in cases of NVC [23]. In our cohort, 33 patients with NVCs received an excess of 437 total days of IV antibiotics that potentially could have been avoided if these recommendations were followed. Unnecessary postoperative antibiotics can have financial and clinical consequences, as prolonged antibiotic therapy has been associated with extended hospitalization and an increased risk of adverse drug effects [19]. Importantly, all patients included in our study received an infectious diseases consultation during admission. This represents a potential gap in antimicrobial stewardship where an opportunity to decrease antibiotic-associated adverse drug events and overall cost to patients and the healthcare system may exist.

Strengths of this study include comprehensive evaluation of antibiotic management both before and after surgery. This is the first study investigation to evaluate the relationship between the duration of blood culture clearance and PVC. The limitations of this study primarily are due to its retrospective nature and relatively small cohort size. First, there was a low rate of IE relapse (1 case), which meant that we were underpowered to thoroughly evaluate the effects of PVC on clinical outcomes. Second, the number of PVCs limited the number of variables able to be included in our multivariable analysis of PVC risk factors. Third, a sizable portion of patients were placed on suppressive antibiotics after completion of therapy, which may have impacted relapses; however, it is worth noting that the use of suppressive therapy was similar between both groups. Fourth, being a major referral center, our institution cares for many patients who are transferred from outside healthcare centers. Thus, for some, records indicating the exact blood culture clearance date were incomplete, limiting analysis of days of blood culture clearance as a variable within a multivariable analysis. Last, given that the postsurgical management of both cohorts were similar, conclusions related to the impact of valve culture positivity on these outcomes should be interpreted with caution.

## CONCLUSION

Fewer days of preoperative antibiotics and enterococcal species were both associated with PVC on univariate and multivariable analyses. PVC was not associated with higher mortality, and 1 case of IE relapse was observed. Carefully defining “day 1” of antibiotic therapy for IE (eg, day of blood culture clearance vs day of surgery) may represent a potential opportunity for antibiotic stewardship in this complex infection and its management.
